# Investigation of *IL-4, IL-10*, and *HVEM* polymorphisms with esophageal squamous cell carcinoma: a case–control study involving 1929 participants

**DOI:** 10.1042/BSR20193895

**Published:** 2020-08-11

**Authors:** Shuchen Chen, Rui Cao, Chao Liu, Weifeng Tang, Mingqiang Kang

**Affiliations:** 1 Department of Thoracic Surgery, Fujian Medical University Union Hospital, Fuzhou, Fujian Province, China; 2 Department of Cardiothoracic Surgery, Affiliated People’s Hospital of Jiangsu University, Zhenjiang, Jiangsu Province, China; 3 Key Laboratory of the Ministry of Education for Gastrointestinal Cancer, Fujian Medical University, Fuzhou, Fujian Province, China

**Keywords:** Interleukin-4, Interleukin-10, Herpesvirus entry mediator, Polymorphism, Risk, Esophageal cancer

## Abstract

It is believed that an individual’s hereditary factors may be involved in the development of esophageal cancer (EC). The present study recruited 721 esophageal squamous cell carcinoma (ESCC) cases and 1208 controls and explored the roles of single nucleotide polymorphisms (SNPs) in the interleukin-4 (*IL-4*), *IL-10*, and herpesvirus entry mediator (*HVEM*) genes in contributing to ESCC risk. *IL-4, IL-10*, and *HVEM* SNPs were analyzed by employing an SNPscan method. After adjustment for body mass index (BMI), smoking, drinking, age and gender, we identified that the rs2070874 T>C locus in *IL-4* gene decreased the risk of ESCC (CC vs. TT: *P*=0.008; CC vs. TT/TC: *P*=0.010). After a stratified analysis, we suggested that the *IL-4* rs2070874 T>C variants might be a protective factor for ESCC in male, ≥63 years old, never smoking, drinking and BMI < 24 kg/m^2^ subgroups. In addition, we identified that the rs2243263 G>C polymorphism in *IL-4* gene was a risk factor for ESCC development in the BMI ≥ 24 kg/m^2^ subgroup (GC vs. GG: *P*=0.030 and GC/CC vs. GG: *P*=0.018). We identified an association of the *IL-4* rs2070874 T>C SNP with the decreased susceptibility of ESCC in stage I/II subgroup. Finally, we found an association of the *IL-10* rs1800872 T>G SNP with a worse differentiation (TG vs. TT: *P*=0.048 and GG/TG vs. TT: *P*=0.032). In conclusion, the findings indicate a potential importance of *IL-4* rs2070874 T>C, *IL-4* rs2243263 G>C and *IL-10* rs1800872 T>G SNPs in the development of ESCC.

## Introduction

In China, esophageal cancer (EC) is the fourth most frequently diagnosed form of malignant tumor in males and the fifth most commonly diagnosed form in females, approximately 320800 and 157200 cases occurred in 2015, respectively [1]. The incidence of EC in Eastern Asia is in the top five worldwide, including China. Esophageal squamous cell carcinoma (ESCC) is a major histological subtype, accounting for 90% of all EC cases. The complex interaction of economical and environmental conditions with individual’s hereditary factors may lead to EC development [2,3]. The etiology and development of EC is not fully understood, despite many investigations have payed close attention to the importance of immunity [4,5]. Recently, it was hypothesized that some important variants in immune-related genes may influence the susceptibility of ESCC.

Interleukin-4 (IL-4), coded by the *IL-4* gene, is an important regulator of the inflammation pathways. IL-4, a pleiotropic cytokine, may be correlated with survival and growth of lymphocytes [6]. IL-4 is produced by mast cell precursors and by the T-cell thymocyte populations. It is important for B-cell activation, proliferation and differentiation [7]. It is reported that IL-4 is necessary for producing immunoglobulin E and implicated in immune diseases. In the process of innate immune responses, IL-4 may activate M2 macrophage, and then play a specific role. It has anti-inflammatory effect, which is relevant to the development of ESCC. Recently, a number of studies have focused on the relationship of IL-4 with cancer development [8,9]. *IL-4* single nucleotide polymorphisms (SNPs) have also been explored for an association with susceptibility to cancer [10–12]. The rs2070874 T>C, located in the 5′-UTR region of the *IL-4* gene, is an important SNP in cancer development. Some meta-analyses have indicated that *IL-4* rs2070874 may be associated with cancer development in Asian populations [13–15]. Kim et al*.* reported that *IL-4* rs2070874 might affect the role of aspirin in regulating IL-4 expression [16]. Rs2243263 G>C polymorphism is an intron SNP of *IL-4* gene. This intron SNP might play a role in splicing. Although the exact role of this intron SNP is unknown, the associations of *IL-4* rs2243263 G>C SNP with the human disease have been explored. A previous study suggested that *IL-4* rs2243263 was associated with the reverse seroconversion of Hepatitis B virus (HBV) [17]. This SNP was also studied for the relationship of the susceptibility to cancer. A previous report investigated the correlation of the *IL-4* rs2243263 locus with colorectal cancer [18]. Although in this study, a null association was identified. However, Lan et al., in a large simple size study, found that the *IL-4* rs2243263 G>C SNP might increase the susceptibility to non-Hodgkin lymphoma [19]. Currently, the associations of *IL-4* the rs2070874 T>C, and rs2243263 G>C polymorphisms with ESCC development are unknown.

The *IL-10* gene is located in chromosome 1q32.2. IL-10, another immune regulator, serves as an inhibitor of dendritic cells and macrophages [20], and inhibits the production of many inflammatory cytokines (e.g. tumor necrosis factor-α, IL-1, IL-6, IL-12, and others) [21]. IL-10 is a vital anti-inflammatory regulator. After IL-10 combines with its receptor (IL-10R), signal transducer and activator of transcription 3 is triggered, which plays a vital role in anti-apoptosis and proliferation [20]. An investigation found that the up-regulated mRNA expression of the *IL-10* gene and higher serum levels of IL-10 were found among subjects who carried the rs1800896 G-allele [22]. The rs1800872 SNP, a promotor variant, could influence the level of IL-10 protein [23]. Some investigations have suggested that the *IL-10* rs1800896 A>G (−1082) [24] and rs1800872 A>C (−592) [25] variants may influence the susceptibility to ESCC. Of late, a meta-analysis indicated that these IL-10 SNPs increased the risk of EC [26]. However, in this earlier meta-analysis, the sample size was very limited (1883 EC patients and 2857 controls included). The association of the *IL-10* rs1800896 A>G and rs1800872 A>C polymorphisms with EC development should be further studied.

Herpesvirus entry mediator (HVEM), also known as TNFRSF14, plays a major role in the immune response [27–29]. HVEM has been found to be expressed in lymphoid cells, as well as in other cells. A previous study suggested that the HVEM/B- and T-lymphocyte attenuator/lymphotoxin/CD160 network in immune reaction to infection and inflammation could play a bidirectional regulatory role [30]. Several investigations have focused on the role of HVEM in cancer survival [31–33]. Zhu et al*.* reported that higher expression of HVEM may promote apoptosis and herald a good prognosis for bladder cancer patients [34]. Additionally, a previous study has indicated that HVEM is implicated in the development of breast cancer (BC) [35]. A SNP in the *HVEM* gene, the G to A of rs2234167 in the exon region, was found to influence the development of BC [36]. However, the association of *HVEM* rs2234167 G>A SNP with the expression of HVEM is unknown. Recently, Migita et al. found that HVEM is critical for both tumor survival and the escape of the host immune system in ESCC cases [37]. Thus, it could be a useful target for ESCC therapy. To date, investigation has not been performed to identify a relationship of the *HVEM* rs2234167 G>A polymorphism with ESCC susceptibility.

Therefore, in this investigation, the *HVEM* rs2234167, *IL-4* rs2070874 and rs2243263, and *IL-10* rs1800896 and rs1800872 polymorphisms were selected and investigated for their effect on ESCC development in a Chinese Han population.

## Materials and methods

### Subjects

Our case−control study was performed in Fujian Union Hospital (Fuzhou, China) and the No.1 People’s Hospital of Zhenjiang City (Zhenjiang, China). This investigation was approved by Jiangsu University (registration ID: K-20160036-Y) and Fujian Medical University (registration ID: 2016-ZQN-25). Participants were recruited between February 2014 and April 2018. Our study included 721 ESCC cases and 1208 controls. These ESCC patients were histopathologically confirmed and were from 41 to 87 years old. Controls were cancer-free individuals from 40 to 87 years old. The controls were not related to any ESCC case. Using a pre-structured questionnaire, we collected epidemiological data from participants. The ESCC patients and normal controls signed consent forms.

### DNA extraction and genotyping of *HVEM* rs2234167, *IL-4* rs2070874, and rs2243263, and *IL-10* rs1800896 and rs1800872 loci

We collected a blood sample (2 ml) from each participant. DNA was extracted carefully as described in a previous study [38]. Using an SNPscan™ assay (Genesky Biotechologies Inc., Shanghai, China), we determined the genotypes of *HVEM* rs2234167, *IL-4* rs2070874, and rs2243263, and *IL-10* rs1800896 and rs1800872 polymorphisms. To confirm the accuracy of genotyping, 77 samples were selected and re-tested. The genotypes of *HVEM* rs2234167, *IL-4* rs2070874, and rs2243263, and *IL-10* rs1800896 and rs1800872 loci were re-analyzed by another technician. The genotypes of *HVEM* rs2234167, *IL-4* rs2070874, and rs2243263, and *IL-10* rs1800896 and rs1800872 SNPs were unchanged.

### Statistical analysis

The difference in alcohol consumption, body mass index (BMI), gender, cigarette use, and age were tested by using *χ*^2^ test. Mean age was calculated by using a Student’s *t* test. We used a Chi-square test (*χ*^2^) or Fisher’s exact test to determine whether the frequencies of *HVEM* rs2234167, *IL-4* rs2070874, and rs2243263, and *IL-10* rs1800896 and rs1800872 variants in ESCC cases and controls were different. A multivariate logistic regression analysis method was used to calculate the crude and adjusted odds ratios (ORs) and 95% confidence intervals (CIs) (SAS 9.4 software package; SAS Institute Inc., Cary, NC, U.S.A.). The relationship of *HVEM* rs2234167, *IL-4* rs2070874, and rs2243263, and *IL-10* rs1800896 and rs1800872 polymorphisms with ESCC development was determined by ORs and 95% CIs. The statistical significance of all analyses was *P*<0.05 (two-sided). An internet-based Hardy–Weinberg equilibrium (HWE) test (http://ihg.gsf.de/cgi-bin/hw/hwa1.pl) was also harnessed to assess whether the distribution of *HVEM* rs2234167, *IL-4* rs2070874, and rs2243263, and *IL-10* rs1800896 and rs1800872 genotypes could represent the included population.

## Results

### Baseline characteristics

In total, 721 ESCC cases and 1208 controls were recruited (Table 1). Of these ESCC cases, 170 were females and 551 were males, average age was 62.59 ± 8.18 years. In the control group, there were 309 females and 899 males with an average age of 62.92 ± 8.94 years. There was no difference in terms of mean age (*P*=0.413). The categorical variables, age and gender, were well-matched (*P*>0.05). However, the distribution of other categorical variables (e.g. tobacco use, BMI, and drinking status) were significantly different (all *P*<0.001). Among ESCC cases, there were 405 (56.17%) with lymphatic metastasis. The AJCC version 8.0 criteria (2018) was used to determine the ESCC stage; and 328 ESCC cases were stage I/II and 393 were stage III/IV. After genotyping the 1929 participants, the association of *HVEM* rs2234167, *IL-4* rs2070874, and rs2243263, and *IL-10* rs1800896 and rs1800872 genotypes with ESCC risk was assessed.

**Table 1:** Distribution of selected demographic variables and risk factors in ESCC cases and controls

Variable	Cases (*n*=721)	Controls (*n*=1208)	*P*^1^
	*n*	*n*	
**Age (years)**	62.59 ± 8.18	62.92 ± 8.94	0.413
**Age (years)**			0.613
<63	337	579	
≥63	384	629	
**Sex**			0.325
Male	551	899	
Female	170	309	
**Tobacco use**			**<0.001**
Never	342	881	
Ever	379	327	
**Alcohol use**			**<0.001**
Never	502	1,046	
Ever	219	162	
**BMI (kg/m^2^)**			**<0.001**
<24	527	651	
≥24	194	557	
**Lymph node status**			
Positive	405		
Negative	316		
**TMN stage**			
I	143		
II	185		
III	307		
IV	86		
**Grade**			
G1	142		
G2	405		
G3	174		

Bold values are statistically significant (*P*<0.05). Abbreviation: TMN, tumor-lymph node-metastasis.

1 Two-sided *χ*^2^ test and Student’s *t* test.

The minor allele frequencies (MAFs) of *HVEM* rs2234167, *IL-4* rs2070874, and rs2243263, and *IL-10* rs1800896 and rs1800872 loci are shown in Table 2. They are similar to the data of Chinese population. As presented in Table 2, the *HVEM* rs2234167, *IL-4* rs2070874, and rs2243263, and *IL-10* rs1800896 and rs1800872 genotypes in controls accorded with HWE.

**Table 2:** Primary information for the included SNPs

Genotyped polymorphisms	*HVEM* rs2234167 G>A	*IL-4* rs2070874 T>C	*IL-4* rs2243263 G>C	*IL-10* rs1800872 T>G	*IL-10* rs1800896 T>C
Chromosome	1	5	5	1	1
Position_38	2562891	132674018	132677607	206773062	206773552
Region	3′-UTR	5′-UTR	intron_variant	5′-flanking	5′-flanking
MAF^1^ in database (1000g- Chinese Han populatons)	0.058	0.228	0.072	0.286	0.048
MAF in our controls (*n*=1208)	0.036	0.196	0.065	0.324	0.060
*P*-value for HWE^2^ test in our controls	0.239	0.484	0.593	0.825	0.871
% Genotyping value	99.38%	99.38%	99.27%	99.33%	99.22%

1 MAF.

2 HWE.

### Relationship of *HVEM* rs2234167, *IL-4* rs2070874, and rs2243263, and *IL-10* rs1800896 and rs1800872 loci with ESCC

Table 3 shows the *HVEM* rs2234167, *IL-4* rs2070874, and rs2243263, and *IL-10* rs1800896 and rs1800872 genotypes. The frequencies of *IL-4* rs2070874 TT, TC, and CC genotypes were 486 (67.88%), 214 (29.89%), and 16 (2.23%) in ESCC cases and 780 (64.95%), 371 (30.89%), and 50 (4.16%) in controls. When the reference was *IL-4* rs2070874 TT genotype, we found the *IL-4* rs2070874 CC genotype significantly decreased the risk of ESCC (*P*=0.023). When the reference was *IL-4* rs2070874 TT/TC genotype, the *IL-4* rs2070874 CC genotype also significantly decreased the risk of ESCC (*P*=0.028). Adjustment for BMI, smoking, drinking, age and gender, the decreased susceptibility was also identified (CC vs. TT: *P*=0.008; CC vs. TT/TC: *P*=0.010).

**Table 3:** The frequencies of *HVEM* rs2234167, *IL-4* rs2070874, rs2243263, and *IL-10* rs1800896 and rs1800872 polymorphisms in different ESCC subgroups

Genotype	Overall cases (*n*=721)		Stage I/II patients (*n*=328)		Stage III/IV patients (*n*=393)		Controls (*n*=1208)	
	*n*	%	*n*	%	*n*	%	*n*	%
*HVEM* rs2234167 G>A								
GG	668	93.30	302	92.92	366	93.61	1,117	93.01
GA	47	6.56	23	7.08	24	6.14	81	6.74
AA	1	0.14	0	0.0	1	0.26	3	0.25
A allele	49	3.42	23	3.54	26	3.32	87	3.62
*IL-4* rs2070874 T>C								
TT	486	67.88	223	68.62	263	67.26	780	64.95
TC	214	29.89	96	29.54	118	30.18	371	30.89
CC	16	2.23	6	1.85	10	2.56	50	4.16
C allele	246	17.18	108	16.62	138	17.65	471	19.61
*IL-4* rs2243263 G>C								
GG	615	86.13	282	87.04	333	85.38	1,048	87.26
GC	96	13.45	41	12.65	55	14.10	149	12.41
CC	3	0.42	1	0.31	2	0.51	4	0.33
C allele	102	7.14	43	6.64	59	7.56	157	6.54
*IL-10* rs1800872 T>G								
TT	349	48.81	161	49.54	188	48.21	550	45.80
TG	301	42.10	136	41.85	165	42.31	523	43.55
GG	65	9.09	28	8.62	37	9.44	128	10.65
G allele	431	30.14	192	29.54	239	30.64	779	32.43
*IL-10* rs1800896 T>C								
TT	625	87.66	280	86.42	345	88.69	1,061	88.34
TC	84	11.78	42	12.96	42	10.80	136	11.32
CC	4	0.56	2	0.62	2	0.51	4	0.34
C allele	92	6.45	46	7.10	46	5.91	144	6.00

*HVEM* rs2234167, *IL-4* rs2243263 and *IL-10* rs1800896 and rs1800872 genotypes are shown in Table 3. Both crude and adjusted comparisons indicated that *HVEM* rs2234167, *IL-4* rs2243263, and *IL-10* rs1800896 and rs1800872 loci were not associated with the risk of ESCC (Table 4).

**Table 4:** Logistic regression analyses of association of *HVEM* rs2234167, *IL-4* rs2070874, rs2243263 and *IL-10* rs1800896 and rs1800872 polymorphisms with risk of ESCC

Genotype	Overall patients (*n*=721) vs. controls (*n*=1208)				Stage I/II patients (*n*=328) vs. controls (*n*=1208)				Stage III/IV patients (*n*=393) vs. controls (*n*=1208)			
	Crude OR (95% CI)	*P*	Adjusted OR^1^ (95% CI)	*P*	Crude OR (95% CI)	*P*	Adjusted OR^1^ (95% CI)	*P*	Crude OR (95% CI)	*P*	Adjusted OR^1^ (95% CI)	*P*
*HVEM* rs2234167 G>A												
GA vs. GG	0.97 (0.67–1.41)	0.874	0.99 (0.67–1.47)	0.952	1.05 (0.65–1.70)	0.841	1.03 (0.63–1.69)	0.895	0.90 (0.57–1.45)	0.675	0.97 (0.59–1.60)	0.903
AA vs. GG	0.56 (0.06–5.37)	0.613	0.40 (0.04–4.49)	0.459	-	-	-	-	1.02 (0.11–9.81)	0.988	0.73 (0.06–8.96)	0.806
GA/AA vs. GG	0.96 (0.66–1.38)	0.809	0.96 (0.65–1.42)	0.852	1.01 (0.63–1.63)	0.959	1.00 (0.61–1.63)	0.985	0.91 (0.57–1.44)	0.683	0.96 (0.59–1.57)	0.868
AA vs. GG/GA	0.56 (0.06–5.38)	0.615	0.40 (0.04–4.49)	0.460	-	-	-	-	1.02 (0.11–9.87)	0.984	0.73 (0.06–8.98)	0.807
*IL-4* rs2070874 T>C												
TC vs. TT	0.93 (0.76–1.13)	0.456	0.92 (0.75–1.15)	0.473	0.91 (0.69–1.19)	0.468	0.93 (0.70–1.22)	0.586	0.94 (0.74–1.21)	0.647	0.95 (0.72–1.24)	0.687
CC vs. TT	**0.51 (0.29–0.91)**	**0.023**	**0.45 (0.24–0.81)**	**0.008**	**0.42 (0.18–0.99)**	**0.048**	**0.36 (0.15–0.87)**	**0.022**	0.59 (0.30–1.19)	0.140	0.52 (0.25–1.09)	0.083
TC/CC vs. TT	0.88 (0.72–1.07)	0.190	0.86 (0.70–1.06)	0.163	0.85 (0.65–1.10)	0.217	0.85 (0.65–1.11)	0.239	0.90 (0.71–1.15)	0.403	0.89 (0.69–1.16)	0.383
CC vs. TT/TC	**0.53 (0.30–0.93)**	**0.028**	**0.46 (0.25–0.83)**	**0.010**	0.43 (0.18–1.02)	0.055	**0.37 (0.15–0.88)**	**0.025**	0.60 (0.30–1.20)	0.152	0.53 (0.26–1.10)	0.089
*IL-4* rs2243263 G>C												
GC vs. CC	1.10 (0.83–1.45)	0.506	1.08 (0.81–1.45)	0.596	1.02 (0.71–1.48)	0.905	1.03 (0.70–1.51)	0.883	1.16 (0.83–1.62)	0.378	1.15 (0.80–1.64)	0.456
CC vs. GG	1.28 (0.29–5.73)	0.749	1.27 (0.26–6.13)	0.763	0.93 (0.10–8.35)	0.949	0.84 (0.09–8.01)	0.876	1.57 (0.29–8.63)	0.601	1.58 (0.25–9.82)	0.627
GC/CC vs. GG	1.10 (0.84–1.45)	0.481	1.09 (0.82–1.45)	0.569	1.02 (0.71–1.47)	0.915	1.02 (0.70–1.49)	0.904	1.17 (0.84–1.63)	0.342	1.16 (0.81–1.65)	0.418
CC vs. GG/GC	1.26 (0.28–5.66)	0.761	1.26 (0.26–6.06)	0.773	0.93 (0.10–8.32)	0.947	0.83 (0.09–7.98)	0.874	1.54 (0.28–8.46)	0.617	1.55 (0.25–9.64)	0.640
*IL-10* rs1800872 T>G												
TG vs. TT	0.91 (0.75–1.10)	0.327	0.94 (0.76–1.16)	0.549	0.89 (0.69–1.15)	0.368	0.90 (0.69–1.18)	0.458	0.92 (0.73–1.17)	0.514	0.95 (0.73–1.23)	0.696
GG vs. TT	0.80 (0.58–1.11)	0.182	0.81 (0.57–1.14)	0.222	0.75 (0.48–1.17)	0.200	0.76 (0.48–1.19)	0.226	0.85 (0.57–1.26)	0.413	0.87 (0.56–1.33)	0.513
GG/TG vs. TT	0.89 (0.74–1.07)	0.201	0.91 (0.75–1.11)	0.359	0.86 (0.67–1.10)	0.230	0.88 (0.68–1.13)	0.299	0.91 (0.72–1.14)	0.407	0.93 (0.73–1.19)	0.581
GG vs. TT/TG	0.84 (0.61–1.15)	0.271	0.83 (0.60–1.16)	0.273	0.79 (0.52–1.21)	0.282	0.79 (0.51–1.23)	0.296	0.88 (0.60–1.29)	0.510	0.89 (0.59–1.34)	0.572
*IL-10* rs1800896 T>C												
TC vs. TT	1.05 (0.79–1.40)	0.748	1.02 (0.75–1.39)	0.894	1.17 (0.81–1.70)	0.405	1.18 (0.80–1.72)	0.408	0.95(0.66–1.37)	0.783	0.88 (0.59–1.31)	0.532
CC vs. TT	1.70 (0.42–6.81)	0.455	1.71 (0.40–7.33)	0.468	1.90 (0.35–10.41)	0.461	1.71 (0.29–9.92)	0.550	1.54 (0.28–8.43)	0.620	1.63 (0.27–9.70)	0.593
TC/CC vs. TT	1.07 (0.80–1.42)	0.653	1.04 (0.77–1.41)	0.793	1.19 (0.83–1.71)	0.346	1.19 (0.82–1.73)	0.360	0.97 (0.68–1.39)	0.854	0.90 (0.61–1.33)	0.604
CC vs. TT/TC	1.69 (0.42–6.77)	0.460	1.71 (0.40–7.30)	0.469	1.86 (0.34–10.21)	0.474	1.68 (0.29–9.72)	0.565	1.55 (0.28–8.48)	0.615	1.65 (0.28–9.82)	0.584

1 Adjusted for age, sex, smoking status, alcohol use and BMI status. Bold values are statistically significant (*P*<0.05).

Additionally, a subgroup analysis was conducted by ESCC stage. We identified an association between *IL-4* rs2070874 T>C SNP and the decreased susceptibility of ESCC in stage I/II subgroup (CC vs. TT: *P*=0.022; CC vs. TT/TC: *P*=0.025, Table 4). However, this association could not been identified for other SNPs.

### Relationship of *HVEM* rs2234167, *IL-4* rs2070874, and rs2243263, and *IL-10* rs1800896 and rs1800872 loci with ESCC in stratified analyses

In a stratified analysis, the *IL-4* rs2070874 genotypes are listed in Table 5. After an adjustment, we suggested that *IL-4* rs2070874 C allele was a protective factor for ESCC in five subgroups (male subgroup: CC vs. TT: *P*=0.028; CC vs. TT/TC: *P*=0.031; ≥63 years old subgroup: CC vs. TT: *P*=0.026; CC vs. TT/TC: *P*=0.029; never smoking subgroup: CC vs. TT: *P*=0.041; CC/TC vs. TT: *P*=0.013 and TC vs. TT: *P*=0.042; drinking subgroup: CC vs. TT: *P*=0.025; CC vs. TT/TC: *P*=0.024 and BMI < 24 kg/m^2^ subgroup: CC vs. TT: *P*=0.010; CC vs. TT/TC: *P*=0.012). In other subgroups, no association of *L-4* rs2070874 with ESCC risk was found (Table 5).

**Table 5:** Stratified analyses between *IL-4* rs2070874 T>C polymorphism and CRC risk by sex, age, BMI, smoking status, and alcohol consumption

Variable	*IL-4* rs2070874 T>C (case/control)^1^			Adjusted OR^2^ (95% CI); *P*				
	TT	TC	CC	TT	TC	CC	TC /CC	CC vs. (TC/TT)
Sex								
Male	366/578	168/282	12/35	1.00	0.93 (0.73–1.20); *P*: 0.593	**0.45 (0.22–0.92);** ***P*: 0.028**	0.88 (0.69–1.12); *P*: 0.281	**0.46 (0.23–0.93);** ***P*: 0.031**
Female	120/202	46/89	4/15	1.00	0.94 (0.61–1.45); *P*: 0.785	0.44 (0.14–1.37); *P*: 0.154	0.86 (0.57–1.31); *P*: 0.489	0.44 (0.14–1.38); *P*: 0.161
Age (years)								
<63	164/292	84/143	4/18	1.00	0.94 (0.68–1.29); *P*: 0.681	0.50 (0.19–1.31); *P*: 0.160	0.89 (0.65–1.22); *P*: 0.465	0.51 (0.20–1.34); *P*: 0.171
≥63	322/488	130/228	12/32	1.00	0.91 (0.68–1.23); *P*: 0.546	**0.41 (0.19–0.90);** ***P*: 0.026**	0.84 (0.63–1.11); *P*: 0.220	**0.42 (0.20–0.92);** ***P*: 0.029**
Smoking status								
Never	246/564	87/276	7/34	1.00	**0.74 (0.55–0.99);** ***P*: 0.042**	**0.41 (0.18–0.96);** ***P*: 0.041**	**0.70 (0.53–0.93);** ***P*: 0.013**	0.45 (0.20–1.05); *P*: 0.064
Ever	240/216	127/95	9/16	1.00	1.26 (0.90–1.76); *P*: 0.177	0.50 (0.21–1.19); *P*: 0.116	1.15 (0.83–1.58); *P*: 0.404	0.46 (0.20–1.09); *P*: 0.079
Alcohol consumption								
Never	341/675	146/323	13/42	1.00	0.91 (0.71–1.16); *P*: 0.428	0.54 (0.28–1.1.03); *P*: 0.062	0.86 (0.68–1.09); *P*: 0.209	0.55 (0.29–1.06); *P*: 0.074
Ever	145/105	68/48	3/8	1.00	1.01 (0.62–1.63); *P*: 0.979	**0.20 (0.05–0.82);** ***P*: 0.025**	0.88 (0.55–1.39); *P*: 0.570	**0.20 (0.05–0.81);** ***P*: 0.024**
BMI (kg/m^2^)								
<24	356/417	154/196	12/32	1.00	0.92 (0.70–1.20); *P*: 0.517	**0.40 (0.20–0.81);** ***P*: 0.010**	0.84 (0.65–1.08); *P*: 0.179	**0.41 (0.20–0.82);** ***P*: 0.012**
≥24	130/363	60/175	4/18	1.00	0.94 (0.65–1.35); *P*: 0.719	0.59 (0.19–1.822); *P*: 0.359	0.90 (0.63–1.29); *P*: 0.573	0.60 (0.20–1.85); *P*: 0.376

1 For *IL-4* rs2070874 T>C, the genotyping was successful in 716 (99.31%) CRC cases and 1201 (99.42%) controls.

2 Adjusted for multiple comparisons [age, sex, BMI, smoking status and alcohol consumption (besides stratified factors accordingly)] in a logistic regression model.

Bold values are statistically significant (*P*<0.05).

The *IL-4* rs2243263 G>C genotypes in the stratified analysis are listed in Table 6. After adjustment, we identified that *IL-4* rs2243263 G>C polymorphism was a risk factor for ESCC development in the BMI ≥ 24 kg/m^2^ subgroup (GC vs. GG: *P*=0.030 and GC/CC vs. GG: *P*=0.018, Table 6).

**Table 6:** Stratified analyses between *IL-4* rs2243263 G>C polymorphism and CRC risk by sex, age, BMI, smoking status, and alcohol consumption

Variable	*IL-4* rs2243263 G>C (case/control)^1^			Adjusted OR^2^ (95% CI); *P*				
	GG	GC	CC	GG	GC	CC	GC/CC	CC vs. (GC/GG)
Sex								
Male	464/779	79/113	2/3	1.00	1.16 (0.83–1.61); *P*: 0.388	1.22 (0.18–8.29); *P*: 0.838	1.16 (0.84-1.61); *P*: 0.378	1.20 (0.18–8.11); *P*: 0.854
Female	151/269	17/36	1/1	1.00	0.86 (0.46–1.60); *P*: 0.636	1.37 (0.09–22.20); *P*: 0.825	0.88 (0.48–1.61); *P*: 0.674	1.39 (0.09–22.54); *P*: 0.816
Age								
<63	215/392	34/59	1/3	1.00	1.06 (0.69–1.65); *P*: 0.780	0.55 (0.04–7.64); *P*: 0.656	1.05 (0.68–1.61); *P*: 0.836	0.55 (0.04–7.57); *P*: 0.652
≥63	400/656	62/90	2/1	1.00	1.12 (0.76–1.67); *P*: 0.567	1.98 (0.26–14.94); *P*: 0.507	1.14 (0.77–1.69); *P*: 0.503	1.95 (0.26–14.70); *P*: 0.517
Smoking status								
Never	300/763	38/108	1/2	1.00	0.90 (0.60–1.34); *P*: 0.601	0.72 (0.07–7.31); *P*: 0.780	0.89 (0.60–1.33); *P*: 0.577	0.73 (0.07–7.41); *P*: 0.788
Ever	315/285	58/41	2/2	1.00	1.39 (0.89–2.18); *P*: 0.147	2.86 (0.23–35.48); *P*: 0.414	1.42 (0.91–2.21); *P*: 0.121	2.71 (0.22–33.47); *P*: 0.438
Alcohol consumption								
Never	433/903	63/134	3/3	1.00	0.96 (0.69–1.33); *P*: 0.787	2.04 (0.39–10.55); *P*: 0.397	0.98 (0.71–1.36); *P*: 0.901	2.05 (0.40–10.61); *P*: 0.393
Ever	182/145	33/15	0/1	1.00	1.86 (0.93–3.73); *P*: 0.080	-	1.70 (0.86–3.35); *P*: 0.127	-
BMI (kg/m^2^)								
<24	457/553	62/89	3/3	1.00	0.84 (0.59–1.22); *P*: 0.364	0.37 (0.04–3.96); *P*: 0.414	0.83 (0.58-1.19); *P*: 0.307	0.38 (0.04–4.04); *P*: 0.424
≥24	158/495	34/60	0/1	1.00	**1.69 (1.05–1.2.71);** ***P*: 0.030**	5.12 (0.42–62.14); *P*: 0.200	**1.75 (1.10–2.78);** ***P*: 0.018**	4.73 (0.39–57.51); *P*: 0.223

1 For *IL-4* rs2243263 G>C, the genotyping was successful in 714 (99.03%) CRC cases and 1201 (99.42%) controls.

2 Adjusted for multiple comparisons [age, sex, BMI, smoking status and alcohol consumption (besides stratified factors accordingly)] in a logistic regression model.

Bold values are statistically significant (*P*<0.05).

In other stratified analyses, adjustment comparisons suggested that *HVEM* rs2234167, and *IL-10* rs1800872 and rs1800896 loci did not confer a risk of ESCC (data not shown).

### Association of *HVEM* rs2234167, *IL-4* rs2070874, and rs2243263, and *IL-10* rs1800896 and rs1800872 loci with lymphatic metastasis in ESCC cases

Among the 721 ESCC patients, 405 patients had lymphatic metastasis. As presented in Table 7, we found a null association of *HVEM* rs2234167, *IL-4* rs2070874, rs2243263 and *IL-10* rs1800896 and rs1800872 SNPs with different lymph node status.

**Table 7:** Logistic regression analyses of association between *HVEM* rs2234167, *IL-4* rs2070874, rs2243263 and *IL-10* rs1800896 and rs1800872 polymorphisms and lymph node status in ESCC patients

Genotype	Positive (*n*=405)		Negative (*n*=316)		Crude OR (95% CI)	*P*	Adjusted OR^1^ (95% CI)	*P*
	*n*	%	*n*	%				
*HVEM* rs2234167 G>A								
GG	378	93.80	290	92.65	1.00		1.00	
GA	24	5.96	23	7.35	0.80 (0.44–1.45)	0.461	0.81 (0.44–1.49)	0.500
AA	1	0.25	0	0	-	-	-	-
GA + AA	25	6.20	23	7.35	0.83 (0.46–1.50)	0.544	0.84 (0.46–1.53)	0.573
GG+GA	402	99.75	313	100.00	1.00		1.00	
AA	1	0.25	0	0	-	-	-	-
A allele	26	3.23	23	3.67				
*IL-4* rs2070874 T>C								
TT	275	68.24	211	67.41	1.00			
TC	118	29.28	96	30.67	0.94 (0.68–1.30)	0.723	0.91 (0.66–1.27)	0.589
CC	10	2.48	6	1.92	1.28 (0.46–3.57)	0.639	1.29 (0.45–3.70)	0.637
CC+TC	128	31.76	102	32.59	0.96 (0.70–1.32)	0.814	0.93 (0.68–1.29)	0.681
TT+TC	393	97.52	307	98.08	1.00		1.00	
CC	10	2.48	6	1.92	1.30 (0.47–3.62)	0.613	1.33 (0.47–3.79)	0.597
C allele	138	17.12	108	17.25				
*IL-4* rs2243263 G>C								
GG	346	86.07	269	86.22	1.00		1.00	
GC	54	13.42	42	13.46	1.00 (0.65–1.54)	0.999	1.02 (0.65–1.59)	0.932
CC	2	0.50	1	0.32	1.56 (0.14–17.24)	0.719	2.18 (0.19–24.54)	0.529
CG+CC	56	13.93	43	13.78	1.01 (0.66–1.55)	0.955	1.04 (0.67–1.62)	0.851
GG+GC	400	99.50	311	99.68	1.00		1.00	
CC	2	0.50	1	0.32	1.56 (0.14–17.23)	0.719	2.17 (0.19–24.43)	0.531
C allele	58	7.21	44	7.05				
*IL-10* rs1800872 T>G								
TT	195	48.51	154	49.20	1.00			
TG	169	42.04	132	42.17	1.01 (0.74–1.38)	0.944	1.01 (0.73–1.38)	0.968
GG	38	9.45	27	8.63	1.11 (0.65–1.90)	0.700	1.17 (0.68–2.03)	0.576
GG+TG	207	51.49	159	50.80	1.03 (0.77–1.38)	0.854	1.03 (0.76–1.40)	0.828
TT+TG	364	90.55	286	88.54	1.00		1.00	
GG	38	9.45	27	8.63	1.11 (0.66–1.86)	0.703	1.17 (0.69–1.98)	0.570
G allele	245	30.47	186	29.71				
*IL-10* rs1800896 T>C								
TT	356	88.78	269	86.22	1.00			
TC	43	10.72	41	13.14	0.79 (0.50–1.25)	0.318	0.82 (0.51–1.31)	0.404
CC	2	0.50	2	0.64	0.76 (0.11–5.40)	0.780	0.88 (0.12–6.44)	0.897
CC+TC	45	11.22	43	13.78	0.79 (0.51–1.24)	0.303	0.82 (0.52–1.30)	0.402
TT+TC	399	99.50	310	99.36	1.00		1.00	
CC	2	0.50	2	0.64	0.78 (0.11–5.55)	0.801	0.90 (0.12–6.61)	0.918
C allele	47	5.86	45	7.21				

1 Adjusted for age, sex, smoking, alcohol use, and BMI status.

### Association of *HVEM* rs2234167, *IL-4* rs2070874, and rs2243263, and *IL-10* rs1800896 and rs1800872 loci with tumor grade of ESCC cases

As presented in Table 1, 142 patients had well-differentiated tumors, 405 had moderately differentiated, tumors and 174 has poorly differentiated tumors. We found an association of the *IL-10* rs1800872 T>G SNP with a worse differentiation (TG vs. TT: *P*=0.048 and GG/TG vs. TT: *P*=0.032, Table 8).

**Table 8:** Logistic regression analyses of association between *HVEM* rs2234167, *IL-4* rs2070874, rs2243263 and *IL-10* rs1800896 and rs1800872 polymorphisms and grades of ESCC

Genotype	G2+G3 (*n*=579)		G1 (*n*=142)		Crude OR (95% CI)	*P*	Adjusted OR^1^ (95% CI)	*P*
	*n*	%	*n*	%				
*HVEM* rs2234167 G>A								
GG	541	93.44	127	89.44	1.00		1.00	
GA	33	5.70	14	9.86	0.55 (0.29–1.07)	0.076	0.57 (0.30–1.11)	0.099
AA	1	0.17	0	0	-	-	-	-
GA + AA	34	5.87	14	9.86	0.57 (0.30–1.09)	0.091	0.59 (0.31–1.13)	0.112
GG+GA	574	99.14	141	99.30	1.00		1.00	
AA	1	0.17	0	0	-	-	-	-
A allele	35	3.02	14	4.93				
*IL-4* rs2070874 T>C								
TT	391	67.53	95	66.90	1.00			
TC	173	29.88	41	28.87	1.03 (0.68–1.54)	0.905	1.06 (0.70–1.60)	0.785
CC	11	1.90	5	3.52	0.54 (0.18–1.58)	0.256	0.56 (0.19–1.66)	0.296
CC+TC	184	31.78	46	32.39	0.97 (0.66–1.44)	0.887	1.01 (0.68–1.50)	0.981
TT+TC	564	97.41	136	95.77	1.00		1.00	
CC	11	1.92	5	3.52	0.53 (0.18–1.55)	0.247	0.55 (0.19–1.62)	0.278
C allele	195	16.84	51	17.96				
*IL-4* rs2243263 G>C								
GG	493	85.15	122	85.92	1.00		1.00	
GC	78	13.47	18	12.68	1.07 (0.62–1.86)	0.803	1.13 (0.65–1.96)	0.674
CC	2	0.35	1	0.70	0.50 (0.05–5.50)	0.567	0.62 (0.05–7.19)	0.702
CG+CC	80	13.82	19	13.38	1.04 (0.61–1.78)	0.882	1.10 (0.64–1.90)	0.727
GG+GC	571	98.62	140	98.59	1.00		1.00	
CC	2	0.35	1	0.70	0.49 (0.04–5.45)	0.562	0.61 (0.05–7.03)	0.690
C allele	82	7.08	20	7.04				
*IL-10* rs1800872 T>G								
TT	269	46.46	80	56.34	1.00			
TG	250	43.18	51	35.92	1.46 (0.99–2.16)	0.059	**1.49 (1.00–2.21)**	**0.048**
GG	55	9.50	10	7.04	1.64 (0.80–3.36)	0.180	1.59 (0.77–3.27)	0.211
GG+TG	305	52.68	61	42.96	**1.49 (1.03–2.16)**	**0.036**	**1.51 (1.04–2.19)**	**0.032**
TT+TG	519	89.64	131	92.25	1.00		1.00	
GG	55	9.50	10	7.04	1.39 (0.69–2.80)	0.359	1.34 (0.66–2.71)	0.419
G allele	360	31.09	71	25.00				
*IL-10* rs1800896 T>C								
TT	501	86.53	124	87.32	1.00			
TC	68	11.74	16	11.27	1.05 (0.59–1.88)	0.864	1.07 (0.60–1.93)	0.809
CC	3	0.52	1	0.70	0.74 (0.08–7.20)	0.797	0.88 (0.09–8.64)	0.909
CC+TC	71	12.26	17	11.97	1.03 (0.59–1.82)	0.909	1.06 (0.60–1.88)	0.833
TT+TC	569	98.27	140	98.59	1.00		1.00	
CC	3	0.52	1	0.70	0.74 (0.08–7.15)	0.793	0.87 (0.09–8.55)	0.903
C allele	74	6.39	18	6.34				

1 Adjusted for age, sex, smoking, alcohol use, and BMI status.

Bold values are statistically significant (*P*<0.05).

## Discussion

Immunotherapy is altering how we comprehend malignancies and offers new methods to treat them. EC is a representative model of immune and inflammation-related cancer [39]. Recently, some studies indicated that the SNPs in inflammation and immune-related genes might influence the risk of EC [40,41]. In this study, we explored the role of immune-related gene SNPs (*HVEM* rs2234167, *IL-4* rs2070874, and rs2243263, and *IL-10* rs1800896 and rs1800872) to ESCC development. We observed that *IL-4* rs2070874 T>C could decrease a risk to ESCC, even in the stage I/II subgroup. However, in BMI ≥ 24 kg/m^2^ subgroup, *IL-4* rs2243263 G>C might increase the risk of ESCC. We also found an association of the *IL-10* rs1800872 T>G SNP with a worse differentiation.

IL-4 is an important regulator of immune and inflammation pathways. Some reports have suggested that IL-4 levels are higher in untreated ESCC patients than in controls [42–44]. It is considered that IL-4 levels may be implicated in the development of ESCC. The *IL-4* rs2070874 T>C polymorphism is a 5′-UTR SNP. In a high-risk gastric cancer (GC) region, a previous study suggested that rs2070874 C allele in the *IL-4* gene might decrease the susceptibility to GC in a Chinese population [45]. Lu et al*.* reported that the rs2070874 C allele increased the risk of HCC in a male subgroup [46]. However, Chang et al. and Wang et al*.* found that the *IL-4* rs2070874 polymorphism might not influence the susceptibility of cancer in Chinese population [47,48]. In this study, we included 1929 subjects and investigated the correlation of this SNP to ESCC susceptibility. We found that *IL-4* rs2070874 T>C polymorphism seemed to be a protective factor for ESCC development. Our findings were similar to a previous meta-analysis that suggested that the *IL-4* rs2070874 C allele could be associated with a decreased susceptibility of gastrointestinal cancer [14]. A functional study indicated that the *IL-4* rs2070874 allele C could promote a higher level of IL-4 in plasma [49]. IL-4 has an anti-inflammatory effect and may decrease the risk of ESCC by inhibiting the inflammation. FitzGerald et al*.* reported that the *IL-4* rs2070874 allele C could decrease the risk of prostate cancer specific mortality [50]. Consistent with that report, we identified an association between the *IL-4* rs2070874 T>C SNP and a decreased susceptibility to ESCC in the stage I/II subgroup. However, we did not find an association of *IL-4* rs2070874 T>C polymorphism with lymphatic metastasis. This might be due to the limited sample sizes. In the future, the relationship of the rs2070874 SNP in *IL-4* gene with progress and prognosis should be further explored.

Rs2243263 G>C, an intron SNP in the *IL-4* gene, was studied for the relationship of this SNP to some diseases. This SNP might decrease the risk of asthma in the African American children, while this relationship was not identified in Caucasians [51]. Hsiao et al*.* reported that the *IL-4* rs2243263 C allele was a protective factor for HBV surface antigen reverse seroconversion in non-Hodgkin lymphoma cases undergoing rituximab treatment. A previous study investigated the relationship of *IL-4* rs2243263 G>C with colon and rectal cancer risk [18], but no association was found. However, in a large simple size study, Lan et al*.* found that the *IL-4* rs2243263 G>C SNP increased the susceptibility to non-Hodgkin lymphoma [19]. In this study, we found that the *IL-4* rs2243263 G>C might increase the risk of ESCC in obese and overweight subjects (Table 6). It was reported that the IL-4 level in mothers was inversely linked to overweight in early childhood and might influence the metabolic profile of childhood [52]. In addition, the level of IL-4 decreased with antipsychotic-induced weight gain [53]. It is suggested that the level of IL-4 could influence obesity and overweight. Introns are regulatory sequences that can affect the expression of genes. Here, we found that the rs2243263 G>C polymorphism, a SNP in *IL-4* intron region, might alter the risk of ESCC. It is presumed that the rs2243263 G>C polymorphism influences the level of IL-4 by regulating gene transcription. In the future, a functional study should be considered to explore the potential mechanism.

The *IL-10* rs1800872 T>G is a promotor SNP. Torres-Poveda et al*.* reported that the expression of *IL-10* mRNA and the level of serum IL-10 were significantly higher in subjects with the *IL-10* rs1800872 T allele [54]. A recent study found that *IL-10* rs1800872 T>G SNP promoted the risk of EC [25]. A meta-analysis also confirmed this association [55]. In our case–control study, we did not find the association of *IL-10* rs1800872 T>G SNP with the development of EC, even in stratified analyses and reviewing different lymph node status. Additionally, Liu et al*.* reported that *IL-10* rs1800872 GG genotypes predicted the worse survival of diffuse large B-cell lymphoma patients treated with rituximab-CHOP (cyclophosphamide, doxorubicin, vincristine, and prednisone) [56]. In this study, we found that the *IL-10* rs1800872 G allele was associated with poorly differentiated tumor. Thus, in the future, the association of the *IL-10* rs1800872 T>G SNP and the survival of ESCC cases should be further studied.

Limitations in the present study should be acknowledged. First, in the present study, we only included five functional SNPs and explored the association of the risk to ESCC. Second, there were other environmental risk factors (e.g. vegetable and fruit intake, aspirin and NSAIDs use, and physical exercise), which we did not consider for their influence to the development of ESCC. Third, the number of ESCC patients was limited and our study may be under-powered in some subgroups. Fourth, in this investigation, the protein expression levels of the suspect factors were not measured. Finally, considering the low penetrance of SNP, the other functional polymorphisms in the *HVEM, IL-4*, and *IL-10* genes should not be ignored.

In summary, the present study suggests that the *IL-4* rs2070874 T>C polymorphism is a protective factor for ESCC development, while the *IL-4* rs2243263 G>C increases a risk to ESCC in obese and overweight subjects. Additionally, it is highlighted that the *IL-10* rs1800872 G allele is associated with poorly differentiated tumor.

## Abbreviations

AJCC: American Joint Committee on Cancer
BC: breast cancer
BMI: body mass index
CI: confidence interval
EC: esophageal cancer
ESCC: esophageal squamous cell carcinoma
GC: gastric cancer
HBV: Hepatitis B virus
HVEM: herpesvirus entry mediator
HWE: Hardy–Weinberg equilibrium
IL: interleukin
NSAID: nonsteroidal anti-inflammatory drug
OR: odds ratio
SNP: single nucleotide polymorphism
